# The pattern of health insurance economic resilience in the Covid 19 pandemic shock

**DOI:** 10.1186/s13104-021-05779-2

**Published:** 2021-09-23

**Authors:** Erfan Kharazmi, Shima Bordbar, Hanie Gholampoor

**Affiliations:** 1 Health Human Resources Research Center, School of Health Management and Information Sciences, Shiraz University of Medical Sciences, Qasr Al-Dasht St., Alley 29, Between Palestine St. and Mulla Sadra, Diamond Building, 7133654361 Shiraz, Iran; 2 Health Economic, Tehran University of Medical Sciences, Tehran, Iran

**Keywords:** Health system, Insurance, Economic resilience, Covid 19

## Abstract

**Objective:**

Health insurance is based on people’s significant risks in receiving health services that they cannot afford alone. Since the outbreak of the corona epidemic, the health insurance system has suffered many economic problems. Designing a model of a health insurance system based on the requirements of a resilient economy can improve the functions of this system in the corona crisis.

**Results:**

In this research 12, structural components were obtained in the form of 4 conceptual components. The 4 main conceptual components are Knowledge-based economy, Economic stability, Economic resilience, and justice. The knowledge-based economy is the basis for the formation of economic resilience in the health insurance systems. Health insurance systems will achieve two crucial intermediate results, namely economic resilience, and economic stability, by building the basic infrastructure of a knowledge-based economy. In the long run, maintaining such intermediate results is the foundation of justice in the health insurance system.

**Supplementary Information:**

The online version contains supplementary material available at 10.1186/s13104-021-05779-2.

## Introduction

The World Health Organization considers the right to health as a fundamental right for all [1, 2]. Countries are required to ensure the availability of sustainable financial resources alongside other health resources to realize this right [3–5]. Insurance usually plays the role of an intermediary organization in transferring financial resources from the consumer of health services to its provider [6, 7]. Health insurance is based on people’s significant risks in receiving health services that they cannot afford alone. One of the goals of health insurance is to distribute income in favor of people who cannot cope with these risks [8, 9].

On the other hand, we live in a century when the economies of the world's countries face various threats and pressures. Each organization reacts differently to stressors according to its position [10–12]. “Economic resilience” has been introduced as a suitable model for overcoming the general challenges of economic systems in recent years [13, 14]. Briguglio uses the idea of “Economic resilience” in two general senses: The first is the ability of the economy to emerge from nasty economic shocks, and the second is the ability of the economy to withstand the effects of these shocks [15, 16].

Health insurance systems are also exposed to economic pressures and damage like other economic sectors. One of the damages that have recently severely affected health systems is the occurrence of the Corona pandemic. Health systems and related insurance systems have been involved in many ways as a result of this pandemic. These effects have not only been in the field of essential services of these systems but have also drastically changed the outputs and consequences of these systems [17–20].

There is a combination of public and private health insurance programs in the Iranian health system. “Social Security Insurance” and “Health Insurance” are the main insurance organizations in Iran. The Armed Forces Insurance Organization and the IMDAD Committee Insurance Organization also operate in Iran. The last two organizations cover a smaller population [21]. Iran, among different countries, in addition to severe and risky exposure to Covid 19 disease, has been exposed to various pressures and injuries from the economic point of view for some time. The present research has studied the model of health insurance systems based on Economic resilience that can maintain the stability of insurance services in Iran.

## Main text

### Method

The concepts of Economic resilience in health insurance were extracted using the opinion of experts in the field of health insurance at the beginning of a descriptive and analytical combined study. However, a list of concepts of Economic resilience in health systems, especially in health insurance, was prepared to help experts by referring to studies. Then the semi-structured interview method was used. In this study, researchers have used a questionnaire developed in Heidari's research [22]. The collective views of 30 key people and experts in the field of Economic resilience and health insurance were extracted, including former and current ministers and deputies of the Ministry of Health, former and current heads and deputies of health insurance organizations, professors of health services management and professors of health economics.

The abovementioned individuals were selected based on the purposive sampling method using the following criteria:

- At least ten years of executive and managerial experience at the level of health insurance or the Ministry of Health
- Membership in the headquarters of the Economic resilience or similar cases in health insurance or the Ministry of Health
- Acquisition of a study and research background on the subject of Economic resilience and insurance systems

People who were reluctant to participate in the study or challenging to reach due to distance or busy schedules were excluded from the study and replaced by new ones. Achieving the final concepts of Economic resilience was done using the content analysis method, and sampling and collecting experts' opinions continued until reaching theoretical saturation of data and not achieving a new theme. The six-step approach of the Clarke & Braun was used, including familiarity with the data, creating the initial code, searching for themes, forming sub-themes, defining and naming the main themes, and preparing a final list at this stage of the research. The last themes were reviewed and validated using the opinion of scientific experts outside the research sample. After identifying and grouping the practical components in the Economic resilience of health insurance systems, Mick Mac's analysis was used to determine the position, influence, dependency, and stability of the designed system. The identified components were scored using a range of numbers from 0 to 3 by a group of experts. Mick Mac matrices were formed in the following four modes: MDI, MII, MPDI, MPII. Then the matrix outputs were categorized and analyzed in the form of graphs and Mick Mac maps.

It was also investigated to calculate the vitality coefficient of each component based on the following formula for more detailed analysis based on the assumptions of the DSM (Design Structure Matrix) technique.

$${\text{Vitality}} = {\text{influence value}}*{\text{dependence value}}$$

### Results

In the first steps of this research, the general components of resistance economy in health insurance systems were identified based on previous studies and expert opinions at three levels of conceptual, structural, and functional features. This study aimed to determine the macro model of the health insurance economic resilience in the critical situation of Covid 19. Therefore, the third-level components were not included in Mick Mac’s analysis. The identified features were summarized as follows (Table 1):

**Table 1:** Dimensions/components of the first and second stages of the health insurance economic resilience in the situation of the Covid 19

Level 1: comprehensive (conceptual) components—title	Level 2: organizing components (structural)—title	Level 3: fundamentals components (functional)—frequency
Knowledge-based economy	Empowerment of private and supplementary insurance	15
	Commercialization	45
	improving the competitive environment	50
	Research and Development	55
Justice	social justice (group)	62
	Deprivation (individual)	54
Economic stability	Sustainable employment	45
	Endogenous financing	56
	Internal self-reliance	60
Economic resilience	Modify consumption patterns	81
	Strengthen the purchasing power of people/patients	65
	Modification of structures	48

Additional file 1: Table S1 shows the consumption pattern modification component has the highest number of essential elements among the organizing components, and the private sector strengthening component has the lowest fundamental part.

The position of each component to the other features and their role was determined by entering the second-level parts in Mick Mac software. The structural modification component has the most direct and indirect influence, among other features. The components of social justice and deprivation are much more dependent than other components.

The direct influence maps of the components indicate that the independent variables are research and development, structural modification, and modification of consumption patterns.

**Fig. 1:**
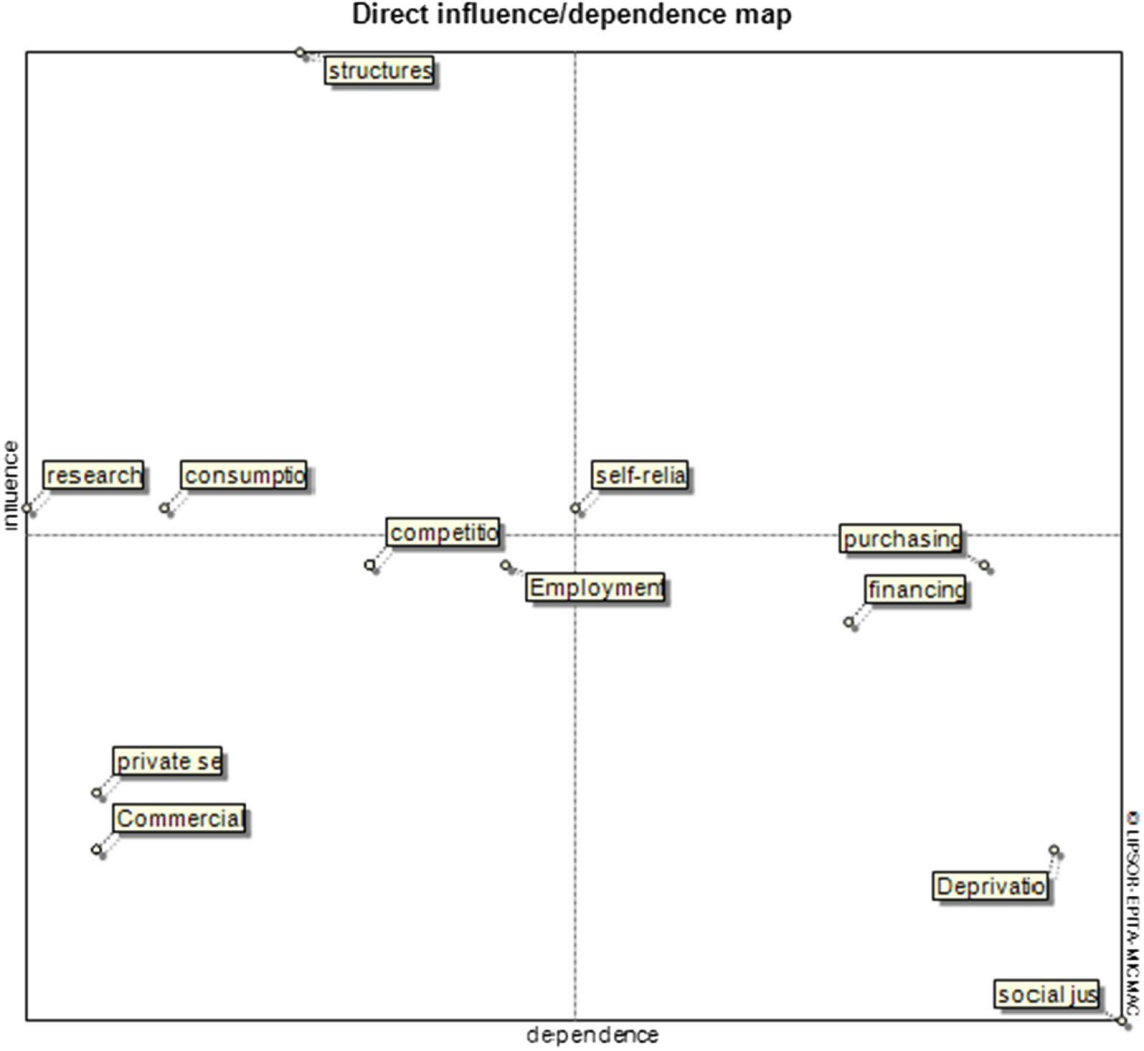
Direct and influence/dependence map of the health insurance economic resilience system

On the other hand, dependent variables and outputs of the economic resilience system are considered health insurance components of people's purchasing power, endogenous financing, deprivation, and social justice. In the model, no component was identified as a linkage variable (Fig. 1).

**Fig. 2:**
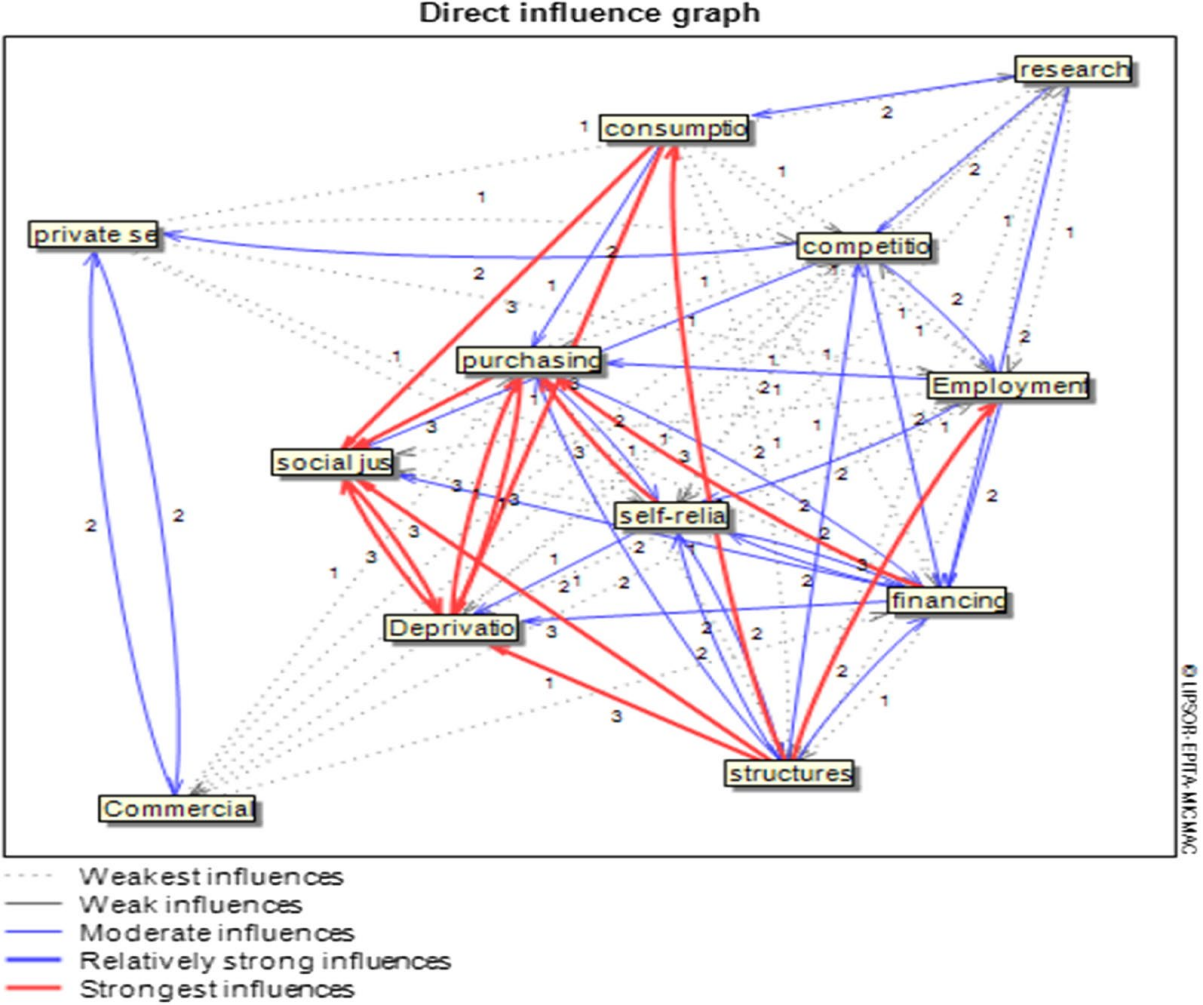
direct influence graph in the health insurance economic resilience system

In Fig. 2, we can see the intensity of the influence of the components on each other. The indirect influence graph of the components clearly shows the strong relationship between structural modification by deprivation and social justice.

### Discussion

Many researchers have considered an issue in recent years because of the need for a local model for health insurance in the event of various crises, including economic concerns and epidemiology [23–26]. Combining the two analyses of content analysis and Mick Mac analysis can be very effective in modeling complex systems [27–31]. The study aimed to identify appropriate inputs to identify influential variables in the model of the health insurance economic resilience systems using the results of the content analysis method. Then, the position and relationship of these variables are examined using the Mick Mac analysis method.

Three variables set in the second quarter of Mick Mac's direct influence map include Research and Development, consumption pattern modification, and structure modification. These variables are considered the inputs of the system and are affected mainly from outside. Therefore, they can be named as environmental and uncontrollable variables. The occurrence of changes in these three variables can affect the system set under review, and in some cases lead to a crisis in the scheme of the health insurance. The structural modification variable is located above the central diameter of the second quarter, so this variable has a more significant influence on the insurance system than the other two variables of this quarter. The structural modification could strengthen health insurance against environmental tensions and crises, including the Corona epidemic, and guarantee continued services in such circumstances [32, 33].

In the third quarter of the map are the variables of empowering the private sector, improving the competitive environment, sustainable employment, and commercialization. These variables are considered independent variables. However, the two variables of sustainable employment and improving the competitive environment can play a regulating role in the health insurance system, given that they are located near the coordinate center. Other sectors of the health insurance economic resilience can perform better by strengthening these two variables. On the one hand, efforts to improve services in the health insurance system will increase with competition between private insurers and innovation will be formed in different parts of health insurance [34, 35].

The fourth quarter includes four variables: strengthening people's purchasing power, endogenous financing, deprivation, and social justice. These variables are affected by other variables and are considered the outputs of the health insurance economic resilience system. That is, the occurrence of any event in other variables can change these four variables. The two variables of deprivation and social justice are the ultimate goal of the health insurance system, which are placed at the bottom end of the fourth quarter.

Additional file 2: Figure S1 shows the two variables of strengthening people's purchasing power and endogenous financing are among the most vital variables of this system. They have a high capacity for influence and dependence at the same time. Health insurance companies must have strategies for endogenous financing to continue and maintain service continuity in the event of various crises. Otherwise, economic resilience in health insurance is problematic [36, 37].

No variables were identified in the first quarter of the map in this study, and the map is L-shaped. Therefore, the current system of economic resilience is a stable system with the studied variables. Analysis of variables in the indirect influence map has similar results, but the position of some variables also shows changes. Additional file 3: Figure S2 shows, the endogenous financing variable is located close to the coordinate center in this case. It has become a regulator. An indirect influence map can also help understand the role and interaction of variables in the long run. For example, the consumption pattern correction variable has lost its regulatory properties in the long run and has become an independent variable. This change of position indicates that in addition to health systems, insurance systems should also be responsible for improving the consumption pattern of health services and constantly monitoring and controlling patients' and society's consumption patterns [38].

Mick Mac's analysis graph shows the intensity of influences between variables in both direct and indirect modes. It can be seen how the variables of strengthening people's purchasing power, deprivation, and social justice are affected by other variables. Additional file 4: Figure S3 shows the only variable of structural modification has retained its strong influence on the two variables of deprivation and social justice in the indirect graph. Here, the importance of variable structural modification in a sustainable insurance system is evident. The decrease in density and the number of red lines of decisive influence in changing the direct to indirect graph show that the resilience economy method is mortal in the long run. It means if there is no planning and effort to maintain this system, the effects of variables on each other will gradually decrease, and the achievement of output (justice) will reduce. Therefore, health insurance systems must continuously monitor and control their economic systems and plan and maintain their essential functions.

Health insurance organizations must pay close attention to their organizational structures to maintain their efficiency in times of crisis such as Covid 19. By reforming organizational structures, it will be possible to achieve "endogenous financing" and "internal self-reliance" in insurance systems. These three factors will ultimately lead to social and individual justice in insurance systems. The existence of a stable resistance economy in insurance companies not only prepares them for the crisis, but also causes them to perform better after the crisis.

Limitation.

- One of the main limitations of this research was access to experts. Many experts were reluctant to be interviewed due to their busy schedules.
- Also due to the prevalence of Covid 19, face-to-face interviews were not possible.

## Supplementary Information

**Additional file 1:** Influence/Dependence Matrix of health insurance factors in economic resilience.

**Additional file 2:** Critical coefficient of components of the health insurance economic resilience systems.

**Additional file 3:** Indirect influence map in the health insurance economic resilience system.

**Additional file 4:** Indirect influence graph in the health insurance economic resilience system.

## Data Availability

The present research data will be available upon logical request. If anyone wants to request data, they should contact corresponding author.

## Abbreviations

MDI: Matrix of direct influences
MII: Matrix of indirect influences
MPDI: Matrix of potential direct influences
MPII: Matrix of potential indirect influences
DSM: Design structure matrix
