# Supplementary material for: The pattern of health insurance economic resilience in the Covid 19 pandemic shock
Source: BMC Res Notes. 2021 Sep 23;14:371. doi: 10.1186/s13104-021-05779-2 (PMC8460182; doi:10.1186/s13104-021-05779-2)
Supplement: Supplementary file 1 — Additional file 1: Influence/Dependence Matrix of health insurance factors in economic resilience. [file 13104_2021_5779_MOESM1_ESM.docx]

**Additional file 1: Table S1 -** **Influence/Dependence Matrix of health insurance factors in economic resilience**

| **n** | **Label** | **Direct Influence** | **Direct Dependence** | **Indirect Influence** | **Indirect Dependence** |
| --- | --- | --- | --- | --- | --- |
| 1 | Structures Modification | 1652 | 578 | 1639 | 649 |
| 2 | Research and development | 991 | 247 | 1114 | 199 |
| 3 | Internal self-reliance | 991 | 909 | 1165 | 981 |
| 4 | Consumption patterns Modification | 991 | 413 | 842 | 263 |
| 5 | Competition environment empowering | 909 | 661 | 869 | 325 |
| 6 | Stable employment | 909 | 826 | 1083 | 505 |
| 7 | Strengthen people's purchasing power | 909 | 1404 | 809 | 1714 |
| 8 | Endogenous financing | 826 | 1239 | 832 | 1071 |
| 9 | Private sector empowering | 578 | 330 | 667 | 122 |
| 10 | Commercialization | 495 | 330 | 526 | 115 |
| 11 | Deprivation | 495 | 1487 | 324 | 2041 |
| 12 | Social justice | 247 | 1570 | 124 | 2009 |
