# Supplementary material for: The pattern of health insurance economic resilience in the Covid 19 pandemic shock
Source: BMC Res Notes. 2021 Sep 23;14:371. doi: 10.1186/s13104-021-05779-2 (PMC8460182; doi:10.1186/s13104-021-05779-2)
Supplement: Supplementary file 2 — Additional file 2: Critical coefficient of components of the health insurance economic resilience systems. [file 13104_2021_5779_MOESM2_ESM.docx]

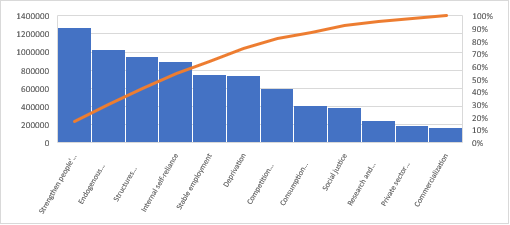


**Additional file 2: Figure S1** Critical coefficient of components of the health insurance economic resilience systems
